# Surgical choice for patients with stage I non-small-cell lung cancer ≤2 cm: an analysis from surveillance, epidemiology, and end results database

**DOI:** 10.1186/s13019-021-01568-x

**Published:** 2021-07-07

**Authors:** Liang Wu, Weigang Zhao, Tangbing Chen, Yi Yang

**Affiliations:** 1 Department of Thoracic Surgery, Shanghai General Hospital, Shanghai Jiao Tong University School of Medicine, Shanghai, 200080 China; 2 Department of Thoracic Surgery, Shanghai Jiao Tong University Affiliated Sixth People’s Hospital, Shanghai, 200233 China; 3 Department of Thoracic Surgery, Shanghai Chest Hospital, Shanghai Jiao Tong University, Shanghai, 200030 China; 4 Department of Thoracic Surgery, Shanghai Jiao Tong University Affiliated Sixth People’s Hospital, Shanghai, 200030 China

**Keywords:** Surgical choice, Non-small-cell lung cancer, Lymph node dissection

## Abstract

**Background:**

No consensus was reached on the surgical procedure for patients with stage I non-small-cell lung cancer (NSCLC) ≤ 2 cm. The aim of this study is to investigate the appropriate surgical procedure for stage I NSCLC ≤2 cm.

**Methods:**

Patients with stage I NSCLC ≤2 cm received wedge resection, segmentectomy, lobectomy between January 2004 and December 2015 were identified using the Surveillance, Epidemiology, and End Results (SEER) database. Data were stratified by age, gender, race, side, location, grade, histology, extent of lymphadenectomy. Overall survival (OS) and lung cancer-specific survival (LCSS) were compared among patients received wedge resection, segmentectomy, lobectomy. Univariate analysis and multivariable Cox regression were performed to identify the prognostic factors of OS and LCSS.

**Results:**

A total of 16,511 patients with stage I NSCLC ≤2 cm were included in this study, of whom 2945 patients were classified as stage I NSCLC ≤1 cm. Lobectomy had better OS and LCSS when compared with wedge resection in patients with NSCLC ≤2 cm. Only OS favored lobectomy compared with segmentectomy in stage I NSCLC>1 to 2 cm. Multivariable analysis showed that segmentectomy had similar OS and LCSS compared with lobectomy in patients with stage I NSCLC ≤2 cm. Lymph node dissection (LND) was associated with better OS in patients with NSCLC ≤2 cm and better LCSS in patients with stage I NSCLC>1 to 2 cm.

**Conclusions:**

Segmentectomy showed comparable survival compared with lobectomy in patients with stage I NSCLC ≤2 cm. LND can provide more accurate pathological stage, may affect survival, and should be recommended for above patients.

## Introduction

Lung cancer is the leading cause of cancer death, and approximately 85% of all diagnoses are non-small-cell lung cancer (NSCLC) [1, 2]. With the introduction of thin-section and low-dose computed tomography (CT) screening for lung cancer, lung cancers are being detected at earlier stages and smaller sizes than before [3]. Although lobectomy is generally accepted as the standard treatment for stage I NSCLC ≤3 cm [4], no consensus on extent of lung resection is reached for stage I NSCLC ≤2 cm.

Beside extent of lung resection, the extent of lymphadenectomy is also controversial in early stage NSCLC. It is generally accepted that systematic nodal dissection can provide more accurate pathological stage and influence the indication of adjuvant treatment which may affect survival. Some surgeons believed that systematic nodal dissection is important for NSCLC even in early stage patients since it can improve survival [5, 6]. On the other hand, some surgeons concluded that systematic mediastinal lymph node dissection is not necessary for clinically evaluated peripheral non-small-cell carcinomas smaller than 2 cm in diameter since it cannot improve survival [7].

Several studies compared the survival between lobectomy and sublobar resection for stage I NSCLC ≤1 cm using SEER database [8–10]. Two studies showed lobectomy had comparable OS and LCSS compared with sublobar resection [8, 10], while one study [9] showed better OS and LCSS for lobectomy compared with subobar resection in stage I NSCLC ≤1 cm. However, there are some limitations in these studies should not be ignored. First, two studies included many patients in early years which may not suitable to generalize to patients nowadays. Second, the status of lymphadenectomy was not analyzed in theses study.

In this study, we used the most recently published SEER database to compare the survival of patients with stage I NSCLC ≤2 cm after sublobar resection and lobectomy. The extent of lymphadenectomy was analyzed in this study.

## Methods

Patients were selected from the SEER database, which contains data on cancer occurrences in 18 geographically diverse populations that represent rural, urban, and regional populations and includes data on cancer occurrences for approximately 30% of the US population [11]. Inclusion criteria: pathologically confirmed primary T1aN0M0 NSCLC ≤2 cm between January 2004 and December 2015; history of wedge resection, segmentectomy, or lobectomy. The demographics of patients, characteristics of lesions, and treatment information were collected and analyzed. Surgical procedures were divided into four groups: lobectomy without LND, lobectomy with LND, segmentectomy, and wedge resection groups. Overall survival (OS) and lung cancer–specific survival (LCSS) information were also collected from SEER database. Overall survival (OS) was calculated from the date of surgery to the date of death from any cause or the date of patients were censored during follow-up, and LCSS was defined as the time from surgery until death as a result of lung cancer.

Categoric variables were compared with the Pearson’s χ2 test. Kaplan- Meier method and log-rank test were performed to estimate and compare the OS and LCSS among lobectomy without LND, lobectomy with LND, segmentectomy, and wedge resection groups by tumor size. Multivariable cox regression was used to determine whether age, gender, race, location, grade, histology, surgical type and LND were associated with the OS and/or LCSS. A two-sided *P* values less than 0.05 were considered statistically significant. All statistics were performed by SPSS version 23.0 (SPSS Inc., Chicago, IL, USA). The survival curve was drawn with MedCalc version 13.

## Results

A total of 16,511 patients with primary T1aN0M0 NSCLC ≤2 cm were included, of whom 2945 patients were ≤ 2 cm and 13,566 patients were>1 to 2 cm. There were 11,773 patients received lobectomy and 4738 patients received sublobar resection. The baseline characteristics of patients and lesions were listed in Table 1 (NSCLC ≤1 cm) and Table 2 (NSCLC>1 to 2 cm). More elderly patients received wedge resection and segmentectomy than lobectomy in both NSCLC ≤1 cm and NSCLC>1 to 2 cm groups. No statistical significance were observed in gender between three surgical procedures in both NSCLC ≤1 cm and NSCLC>1 to 2 cm groups.

**Table 1:** Characteristics of patients with stage IA non–small-cell lung cancer ≤1 cm

Characteristic	Lobectomy (*N* = 1768)	Segmentectomy (*N* = 182)	Wedge resection (*N* = 995)	*P*
Age				< 0.001
< 60 y	484 (27)	30 (16)	188 (19)	
60–74 y	1012 (57)	109 (60)	588 (59)	
≥ 75y	272 (16)	43 (24)	219 (22)	
Gender				0.877
Male	675 (38)	73 (40)	381 (38)	
Female	1093 (62)	109 (60)	614 (62)	
Race				
White	1470 (83)	155 (85)	812 (82)	0.890
Black	133 (7)	12 (7)	80 (8)	
Hispanic	68 (4)	6 (3)	39 (4)	
Asian	82 (5)	9 (5)	54 (5)	
Other	15 (1)	0 (0)	10 (1)	
Side				< 0.001
Left	647 (37)	93 (51)	412 (41)	
Right	1121 (63)	89 (49)	583 (59)	
Location				0.003
Upper	1135 (64)	113 (62)	650 (65)	
Middle	126 (7)	2 (1)	52 (5)	
Lower	507 (29)	67 (37)	293 (30)	
Grade				0.090
Grade I	521 (29)	52 (28)	310 (31)	
Grade II	705 (40)	78 (43)	340 (34)	
Grade III	338 (19)	31 (17)	206 (21)	
Grade IV	17 (1)	3 (2)	17 (2)	
Unknow	187 (11)	18 (10)	122 (12)	
Histology				0.153
sq	330 (19)	29 (16)	191 (19)	
ad	1053 (59)	119 (65)	570 (57)	
BAC	228 (13)	19 (10)	121 (12)	
Large cell	37 (2)	7 (4)	25 (3)	
Other	120 (7)	8 (4)	88 (9)	

*Abbreviations*: *sq*. Squamous cell carcinoma, *ad* Adenocarcinoma, *BAC* Bronchoalveolar carcinoma

**Table 2:** Characteristics of patients with stage IA non–small-cell lung cancer > 1 to 2 cm

Characteristic	Lobectomy (*N* = 10,005)	Segmentectomy (*N* = 757)	Wedge resection (*N* = 2804)	*P*
Age				< 0.001
< 60 y	2244 (22)	140 (19)	466 (17)	
60–74 y	5695 (56)	395 (52)	1474 (52)	
≥ 75y	2066 (21)	222 (29)	864 (31)	
Gender				0.175
Male	4213 (42)	295 (39)	1198 (43)	
Female	5792 (58)	462 (61)	1606 (57)	
Race				
White	8103 (81)	629 (83)	2334 (83)	< 0.001
Black	751 (7)	64 (9)	227 (8)	
Hispanic	446 (5)	32 (4)	109 (4)	
Asian	638 (6)	31 (4)	118 (4)	
Other	67 (1)	1(0)	16 (1)	
Side				< 0.001
Left	3890 (39)	360 (48)	1228 (44)	
Right	6115 (61)	397 (52)	1576 (56)	
Location				< 0.001
Upper	6322 (63)	438 (58)	1831 (65)	
Middle	660 (7)	20 (3)	123 (4)	
Lower	3023 (30)	299 (39)	850 (30)	
Grade				< 0.001
Grade I	2273 (23)	177 (23)	615 (22)	
Grade II	4561 (45)	346 (46)	1169 (42)	
Grade III	2384 (24)	166 (22)	736 (26)	
Grade IV	115 (1)	6 (1)	48 (2)	
Unknow	672 (7)	62 (8)	236 (8)	
Histology				< 0.001
sq	2000 (20)	174 (23)	707 (25)	
ad	6074 (61)	432 (57)	1489 (53)	
BAC	995 (10)	90 (12)	273 (10)	
Large cell	211 (2)	15 (2)	92 (3)	
Other	725 (7)	46 (6)	243 (9)	

*Abbreviations*: *sq.* Squamous cell carcinoma, *ad* Adenocarcinoma, *BAC* Bronchoalveolar carcinoma

**Fig. 1:**
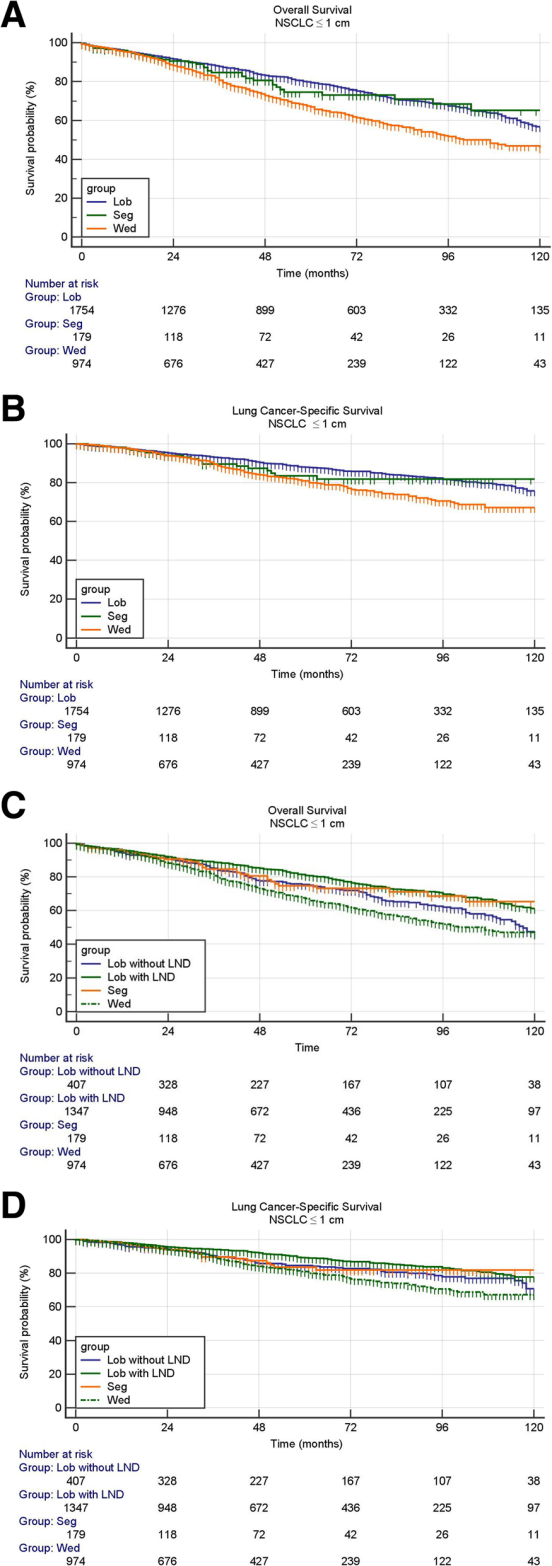
**A** Overall survivals in patients with stage IA non–small-cell lung cancer ≤1 cm undergoing lobectomy, segmentectomy or wedge resection. **B**. Lung cancer-specific survivals in patients with stage IA non–small-cell lung cancer ≤1 cm undergoing lobectomy, segmentectomy or wedge resection. **C**. Overall survivals in patients with stage IA non–small-cell lung cancer ≤1 cm undergoing lobectomy with LND, lobectomy without LND, segmentectomy or wedge resection. **D**. Lung cancer-specific survivals in patients with stage IA non–small-cell lung cancer ≤1 cm undergoing lobectomy with LND, lobectomy without LND, segmentectomy or wedge resection

The survival analysis by log-rank test showed that wedge resection had obviously worse OS (hazard ratio (HR), 1.59; 95% CI, 1.36 to 1.86; *P* < 0.001) and LCSS (HR, 1.58; 95% CI, 1.27 to 1.96; *P* < 0.001) than lobectomy in patients with NSCLC ≤1 cm (Fig. 1A, B, Table 3). However, no survival benefit was observed in lobectomy when compared with segmentectomy in OS (HR, 1.05; 95% CI, 0.74 to 1.48; *P* = 0.798) and LCSS (HR, 1.11; 95% CI, 0.69 to 1.80; *P* = 0.642) for patients with NSCLC ≤1 cm (Fig. 1A, B, Table 3). When patients with lobectomy were divided into lobectomy with LND and lobectomy without LND, lobectomy without LND showed obvious worse OS (HR, 1.40; 95% CI, 1.11 to 1.74; *P* < 0.001) and LCSS (HR, 1.41; 95% CI, 1.04 to 1.91; *P* = 0.018) than lobectomy with LND (Fig. 1C, D, Table 3). Wedge resection still showed worse OS compared with lobectomy without LND (HR, 1.25; 95% CI, 1.03 to 1.52; *P* < 0.001) while no statistical significance was observed in LCSS (HR, 1.24; 95% CI, 0.95 to 1.63; *P* = 0.121) (Fig. 1C, D, Table 3).

**Table 3:** Overall survival and lung cancer-specific survival in patients with stage IA non–small-cell lung cancer ≤1 cm and > 1 to 2 cm groups

Variables	NSCLC ≤1 cm				NSCLC > 1 to 2 cm			
	Overall survival		Lung cancer-specific survival		Overall survival		Lung cancer-specific survival	
	HR (95%)	*P*	HR (95%)	*P*	HR (95%)	*P*	HR (95%)	*P*
Wedge vs. seg	1.52 (1.13–2.04)	0.016	1.40 (0.93–2.11)	0.150	1.52 (1.13–2.04)	0.016	1.51 (1.27–1.80)	< 0.001
Wedge vs. lob without LND	1.25 (1.03–1.52)	0.025	1.24 (0.95–1.63)	0.121	1.63 (1.49–1.78)	< 0.001	1.64 (1.44–1.87)	< 0.001
Wedge vs. lob with LND	1.76 (1.49–2.07)	< 0.001	1.75 (1.40–2.21)	< 0.001	1.90 (1.74–2.06)	< 0.001	1.89 (1.69–2.12)	< 0.001
Wedge vs. lob	1.59 (1.36–1.86)	< 0.001	1.58 (1.27–1.96)	< 0.001	1.82 (1.68–1.97)	< 0.001	1.82 (1.63–2.03)	< 0.001
Seg vs. lob without LND	0.82 (0.58–1.16)	0.287	0.84 (0.53–1.36)	0.513	1.16 (1.00–1.36)	0.041	1.08 (0.88–1.33)	0.460
Seg vs. lob with LND	1.15 (0.79–1.67)	0.414	1.24 (0.75–2.07)	0.360	1.35 (1.15–1.57)	< 0.001	1.26 (1.02–1.56)	0.018
Seg vs. lobe	1.05(0.74–1.48)	0.798	1.11(0.69–1.80)	0.642	1.29 (1.11–1.50)	< 0.001	1.20(0.98–1.48)	0.005
Lob without LND vs. with LND	1.40 (1.12–1.74)	0.001	1.41 (1.04–1.91)	0.018	1.15 (1.06–1.24)	< 0.001	1.15 (1.02–1.29)	0.012

*Abbreviations*: *seg* Segmentectomy, *lob* Lobectomy, *LND* Lymph node dissection

**Fig. 2:**
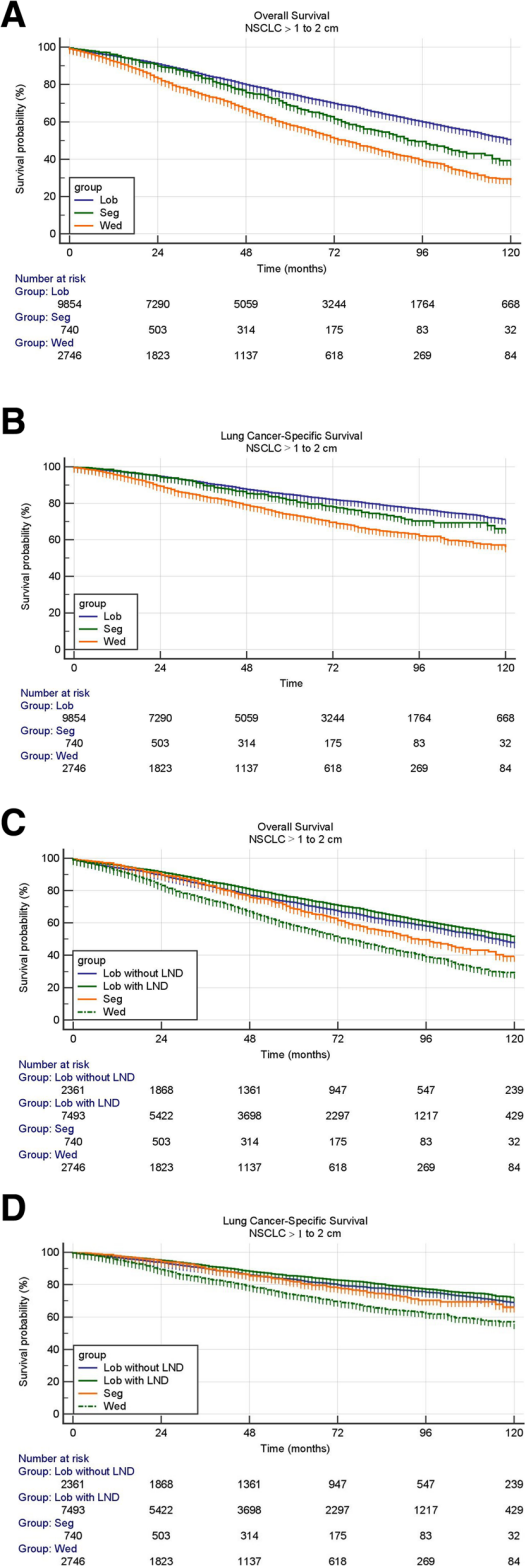
**A** Overall survivals in patients with stage IA non–small-cell lung cancer > 1 to 2 cm undergoing lobectomy, segmentectomy or wedge resection. **B**. Lung cancer-specific survivals in patients with stage IA non–small-cell lung cancer > 1 to 2 cm undergoing lobectomy, segmentectomy or wedge resection. **C**. Overall survivals in patients with stage IA non–small-cell lung cancer > 1 to 2 cm undergoing lobectomy with LND, lobectomy without LND, segmentectomy or wedge resection. **D**. Lung cancer-specific survivals in patients with stage IA non–small-cell lung cancer > 1 to 2 cm undergoing lobectomy with LND, lobectomy without LND, segmentectomy or wedge resection

For patients with NSCLC >1 to 2 cm, both wedge resection and segmentectomy had worse OS (HR, 1.82; 95% CI, 1.68 to 1.97; *P* < 0.001) (HR, 1.29; 95% CI, 1.11 to 1.50; *P* < 0.001) and LCSS (HR, 1.82; 95% CI, 1.63 to 2.03; *P* < 0.001) (HR, 1.20; 95% CI, 0.98 to 1.48; *P* = 0.005) than lobectomy. (Fig. 2A, B, Table 3). When patients with lobectomy were divided into lobectomy with LND and lobectomy without LND, lobectomy without LND showed obvious worse OS (HR, 1.15 95% CI, 1.06 to 1.24; *P* < 0.001) and LCSS (HR, 1.15; 95% CI, 1.02 to 1.29; *P* = 0.012) than lobectomy with LND (Fig. 2C, D, Table 3). Wedge resection still showed worse OS (HR, 1.63; 95% CI, 1.49 to 1.78; *P* < 0.001) and LCSS (HR, 1.64; 95% CI, 1.44 to 1.87; *P* < 0.001) compared with lobectomy without LND (Fig. 2C, D, Table 3). Segmentectomy showed worse OS (HR, 1.16; 95% CI, 1.00to 1.36; *P* = 0.041) and comparable LCSS (HR, 1.08; 95% CI, 0.88 to 1.33; *P* = 0.460) compared with lobectomy without LND (Fig. 2C, D, Table 3).

Univariate analysis showed age, gender, race, grade, histology, surgical type and extent of lymphadenectomy were associated with OS and LCSS in patients with NSCLC ≤1 cm. We then bring factors with *P* value < 0.2 into multivariable cox regression, the results showed age, gender, race, grade, histology, surgical type were still associated with OS and LCSS in patients with NSCLC ≤1 cm. Wedge resection was independent risk factor associated with statistical significant poorer OS (HR, 1.255; 95% CI, 1.022 to 1.540; *P* = 0.012) and borderline significant poor LCSS (HR, 1.292; 95% CI, 0.972 to 1.718; *P* = 0.079) than lobectomy while segmentectomy had comparable OS (HR, 0.817; 95% CI, 0.565 to 1.180; *P* = 0.286) and LCSS (HR, 0.892; 95% CI, 0.5490 to 1.469; *P* = 0.652) compared with lobectomy in patients with NSCLC≤1 cm. LND was independently associated with statistically significant better OS (HR, 1.143; 95% CI, 1.030 to 1.268; *P* = 0.012) and borderline significant better LCSS (HR, 1.134; 95% CI, 0.982 to 1.310; *P* = 0.088) (Table 4).

**Table 4:** Multivariable cox regression for overall survival and lung cancer-specific survival in patients with non-small-cell lung cancer ≤1 cm

Variables	Overall survival				Lung cancer-specific survival			
	Univariate analysis		Multivariable Cox regression		Univariate analysis		Multivariable Cox regression	
	HR (95%)	p	HR (95%)	p	HR (95%)	p	HR (95%)	p
Age								
≤ 60 y	1.000 (reference)		1.000 (reference)		1.000 (reference)		1.000 (reference)	
60–70 y	1.569 (1.286 to 1.913)	< 0.001	1.420 (1.161 to 1.737)	< 0.001	1.224 (0.949 to 1.578)	0.905	1.139 (0.880 to 1.474)	0.325
> 70 y	2.853(2.285 to 3.562)	< 0.001	2.442 (1.943 to 3.068)	< 0.001	1.984 (1.476 to 2.669)	< 0.001	1.753 (1.291 to 2.380)	< 0.001
Gender								
Male	1.000 (reference)		1.000 (reference)		1.000 (reference)		1.000 (reference)	
Female	0.666 (0.577 to 0.767)	< 0.001	0.732 (0.633 to 0.846)	< 0.001	0.707 (0.580 to 0.862)	< 0.001	0.773 (0.632 to 0.945)	0.013
Race								
White	1.000 (reference)		1.000 (reference)		1.000 (reference)		1.000 (reference)	
Black	1.053 (0.811 to 1.367)	0.702	1.134 (0.871 to 1.475)	0.352	1.055 (0.736 to 1.513)	0.772	1.077 (0.749 to 1.548)	0.692
Hispanic	0.471 (0.273 to 0.813)	0.007	0.471 (0.272 to 0.814)	0.007	0.558 (0.278 to 1.120)	0.102	0.556 (0.276 to 1.120)	0.102
Asian	0.418 (0.251 to 0.696)	0.001	0.454 (0.272 to 0.756)	0.003	0.373 (0.177 to 0.785)	0.010	0.407 (0.193 to 0.858)	0.019
Other	0.607 (0.197 to 1.878)	0.389	0.732 (0.236 to 2.269)	0.591	NA	NA	NA	NA
Side								
LEFT	1.000 (reference)		1.000 (reference)		1.000 (reference)		1.000 (reference)	
RIGHT	0.964 (0.834 to 1.114)	0.618	NA	NA	0.967 (0.790 to 1.182)	0.742	NA	NA
Location								
Upper	1.000 (reference)		1.000 (reference)		1.000 (reference)		1.000 (reference)	
Middle	0.689 (0.490 to 0.969)	0.033	0.798 (0.566 to 1.126)	0.201	0.686 (0.427 to 1.103)	0.121	0.786 (0.487 to 1.269)	0.786
Lower	0.918 (0.781 to 1.079)	0.299	0.965 (0.820 to 1.135)	0.667	0.923 (0.738 to 1.155)	0.486	0.979 (0.781 to 1.228)	0.857
Grade								
Grade I	1.000 (reference)		1.000 (reference)		1.000 (reference)		1.000 (reference)	
Grade II	1.903 (1.548 to 2.338)	< 0.001	1.505 (1.211 to 1.870)	< 0.001	2.423 (1.786 to 3.287)	< 0.001	2.036 (1.483 to 2.795)	< 0.001
Grade III	2.373 (1.904 to 2.958)	< 0.001	1.640 (1.291 to 2.084)	< 0.001	2.859 (2.064 to 3.959)	< 0.001	2.089 (1.474 to 2.960)	< 0.001
Grade IV	1.704 (0.965 to 3.010)	0.068	1.000 (0.524 to 1.907)	0.999	1.886 (0.814 to 4.369)	0.141	0.948 (0.374 to 2.407)	0.912
Unknow	1.896 (1.459 to 2.464)	< 0.001	1.806 (1.379 to 2.365)	< 0.001	2.584 (1.784 to 3.744)	< 0.001	2.524 (1.723 to 3.697)	< 0.001
Histology								
sq	1.000 (reference)		1.000 (reference)		1.000 (reference)		1.000 (reference)	
ad	0.598 (0.535 to 0.670)	< 0.001	0.751 (0.628 to 0.898)	0.002	0.777 (0.606 to 0.996)	0.048	0.994 (0.767 to 1.287)	0.963
BAC	0.350 (0.269 to 0.451)	< 0.001	0.479 (0.357 to 0.641)	< 0.001	0.378 (0.254 to 0.560)	< 0.001	0.530 (0.344 to 0.815)	0.004
Large cell	0.947 (0.643 to 1.395)	0.786	1.110 (0.718 to 1.718)	0.640	1.462 (0.892 to 2.395)	0.134	1.793 (1.038 to 3.097)	0.037
Other	0.820 (0.637 to 1.056)	0.127	0.858 (0.660 to 1.117)	0.258	1.046 (0.731 to 1.496)	0.806	1.083 (0.746 to 1.571)	0.677
Surgical type								
Lobectomy	1.000 (reference)		1.000 (reference)		1.000 (reference)		1.000 (reference)	
Segmentectomy	1.046 (0.745 to 1.469)	0.795	0.817 (0.565 to 1.180)	0.286	1.121 (0.710 to 1.770)	0.625	0.891 (0.540 to 1.469)	0.652
Wedge resection	1.593 (1.375 to 1.846)	< 0.001	1.255 (1.022 to 1.540)	0.031	1.587 (1.293 to 1.947)	< 0.001	1.292 (0.972 to 1.718)	0.079
Lymph node								
LND	1.000 (reference)		1.000 (reference)		1.000 (reference)		1.000 (reference)	
Without LND	1.260 (1.170 to 1.356)	< 0.001	1.143 (1.030 to 1.268)	0.012	1.590 (1.296 to 1.953)	< 0.001	1.134 (0.982 to 1.310)	0.088

*Abbreviations*: *sq.* Squamous cell carcinoma, *ad* Adenocarcinoma, *BAC* Bronchoalveolar carcinoma, *LND* Lymph node dissection

Univariate analysis showed age, gender, race, location, grade, histology, surgical type, extent of lymphadenectomy were associated with OS and LCSS in patients with NSCLC>1 to 2 cm. We then bring factors with *P* value < 0.2 into multivariable cox regression, the results showed age, gender, race, grade, histology, surgical type were still associated with OS and LCSS in patients with NSCLC>1 to 2 cm. Wedge resection was independent risk factor associated with poorer OS (HR, 1.499; 95% CI, 1.371 to 1.640; *P* < 0.001) and LCSS (HR, 1.543; 95% CI, 1.365 to 1.744; P < 0.001) than lobectomy while segmentectomy had comparable OS (HR, 1.094; 95% CI, 0.945 to 1.267; *P* = 0.231) and LCSS (HR, 1.051; 95% CI, 0.856 to 1.290; *P* = 0.636) compared with lobectomy in patients with NSCLC>1 to 2 cm. LND was independently associated with statistically significant better OS (HR, 1.066; 95% CI, 1.024 to 1.110; *P* = 0.012) and LCSS (HR, 1.072; 95% CI, 1.014 to 1.133; *P* = 0.015) (Table 5).

**Table 5:** Multivariable cox regression for overall survival and lung cancer-specific survival in patients with non-small-cell lung cancer ≤1 cm

Variables	Overall survival				Lung cancer-specific survival			
	Univariate analysis		Multivariable cox regression		Univariate analysis		Multivariable cox regression	
	HR (95%)	p	HR (95%)	p	HR (95%)	p	HR (95%)	p
Age								
≤ 60 y	1.000 (reference)		1.000 (reference)		1.000 (reference)		1.000 (reference)	
60–70 y	1.622 (1.480 to 1.779)	< 0.001	1.531 (1.395 to 1.680)	< 0.001	1.408 (1.252 to 1.584)	< 0.001	1.350 (1.199 to 1.520)	< 0.001
> 70y	2.793 (2.534 to 3.080)	< 0.001	2.566 (2.323 to 2.835)	< 0.001	1.957 (1.720 to 2.228)	< 0.001	1.836 (1.609 to 2.096)	< 0.001
Gender								
Male	1.000 (reference)		1.000 (reference)		1.000 (reference)		1.000 (reference)	
Female	0.683 (0.643 to 0.725)	< 0.001	0.722 (0.679 to 0.767)	< 0.001	0.731 (0.674 to 0.795)	< 0.001	0.772 (0.710 to 0.839)	< 0.001
Race								
White	1.000 (reference)		1.000 (reference)		1.000 (reference)		1.000 (reference)	
Black	0.990 (0.881 to 1.111)	0.860	1.069 (0.951 to 1.200)	0.266	0.988 (0.844 to 1.157)	0.882	1.030 (0.879 to 1.207)	0.718
Hispanic	0.798 (0.676 to 0.942)	0.081	0.917 (0.776 to 1.082)	0.307	0.803 (0.640 to 1.007)	0.058	0.898 (0.715 to 1.127)	0.355
Asian	0.598 (0.508 to 0.703)	< 0.001	0.682 (0.580 to 0.803)	0.002	0.609 (0.488 to 0.760)	< 0.001	0.695 (0.557 to 0.869)	0.001
Other	0.760 (0.473 to 1.222)	0.260	0.843 (0.524 to 1.355)	0.482	0.751 (0.392 to 1.442)	0.393	0.820 (0.427 to 1.574)	0.552
Side								
LEFT	1.000 (reference)		1.000 (reference)		1.000 (reference)		1.000 (reference)	
RIGHT	0.978 (0.920 to 1.040)	0.486	NA	NA	0.980 (0.902 to 1.067)	0.649	NA	NA
Location								
Upper	1.000 (reference)		1.000 (reference)		1.000 (reference)		1.000 (reference)	
Middle	0.846 (0.738 to 0.970)	0.017	0.905 (0.789 to 1.038)	0.155	0.822 (0.681 to 0.993)	0.043	0.892 (0.738 to 1.079)	0.241
Lower	0.986 (0.922 to 1.054)	0.683	1.019 (0.953 to 1.089)	0.587	0.968 (0.883 to 1.061)	0.488	1.017 (0.927 to 1.114)	0.726
Grade								
Grade I	1.000 (reference)		1.000 (reference)		1.000 (reference)		1.000 (reference)	
Grade II	1.790 (1.630 to 1.967)	< 0.001	1.505 (1.364 to 1.661)	< 0.001	1.987 (1.737 to 2.273)	< 0.001	1.753 (1.524 to 2.015)	< 0.001
Grade III	2.334 (2.115 to 2.574)	< 0.001	1.793 (1.613 to 1.992)	< 0.001	2.705 (2.353 to 3.109)	< 0.001	2.215 (1.909 to 2.569)	< 0.001
Grade IV	2.435 (1.913 to 3.099)	< 0.001	1.447 (1.105 to 1.896)	0.008	2.709 (1.939 to 3.785)	< 0.001	1.695 (1.170 to 2.456)	0.006
Unknow	1.744 (1.520 to 2.001)	< 0.001	1.623 (1.411 to 1.868)	< 0.001	2.042 (1.688 to 2.470)	< 0.001	1.938 (1.596 to 3.353)	< 0.001
Histology								
sq	1.000 (reference)		1.000 (reference)		1.000 (reference)		1.000 (reference)	
ad	0.590 (0.550 to 0.633)	< 0.001	0.775 (0.720 to 0.834)	< 0.001	0.731 (0.661 to 0.807)	< 0.001	0.953 (0.860 to 1.057)	0.364
BAC	0.353 (0.313 to 0.398)	< 0.001	0.514 (0.451 to 0.586)	< 0.001	0.399 (0.336 to 0.474)	< 0.001	0.613 (0.509 to 0.738)	< 0.001
Large cell	1.118 (0.954 to 1.309)	0.170	1.197 (1.003 to 1.428)	0.048	1.370 (1.105 to 1.696)	0.004	1.403 (1.107 to 1.780)	0.005
Other	0.736 (0.657 to 0.825)	< 0.001	0.854 (0.760 to 0.960)	0.009	0.876 (0.748 to 1.026)	0.102	0.984 (0.837 to 1.157)	0.847
Surgical type								
Lobectomy	1.000 (reference)		1.000 (reference)		1.000 (reference)		1.000 (reference)	
segmentectomy	1.290 (1.129 to 1.476)	< 0.001	1.094 (0.945 to 1.267)	0.231	1.205 (0.998 to 1.456)	0.054	1.051 (0.856 to 1.290)	0.636
Wedge resection	1.832 (1.710 to 1.962)	< 0.001	1.499 (1.371 to 1.640)	< 0.001	1.825 (1.663 to 2.004)	< 0.001	1.543 (1.365 to 1.744)	< 0.001
Lymph node								
LND	1.000 (reference)		1.000 (reference)		1.000 (reference)		1.000 (reference)	
Without LND	1.213 (1.177 to 1.251)	< 0.001	1.066 (1.024 to 1.110)	0.002	1.212 (1.163 to 1.263)	< 0.001	1.072 (1.014 to 1.133)	0.015

*Abbreviations*: *sq.* Squamous cell carcinoma, *ad* Adenocarcinoma, *BAC* Bronchoalveolar carcinoma, *LND* Lymph node dissection

## Discussion

In this study, we compared survival results of patients with stage I NSCLC ≤2 cm treated by wedge resection, segmentectomy, lobectomy. Our study showed patients received wedge resection had obviously worse OS and borderline significant worse LCSS than lobectomy in patients with NSCLC ≤1 cm. The OS and LCSS were significant worse in patients receive wedge resection than lobectomy with NSCLC>1 to 2 cm. No statistical significance was observed in OS and LCSS between segmentectomy and lobectomy in both patients with NSCLC ≤1 cm and NSCLC>1 to 2 cm. LND turned out to be an independent risk factor for better OS in patients with NSCLC ≤1 cm. For patients with NSCLC>1 to 2 cm, LND was associated with better OS and LCSS.

With the wider use and higher resolution of computed tomography (CT) screening for lung cancer, more and more early stage lung cancers are being detected. A randomized controlled trial in early years showed lobectomy was superior to limited resection for patients with stage I NSCLC ≤3 cm with lower death rate and locoregional recurrence rate [4]. However, the results of this study may not able to generalize to patients nowadays since the operative skills and histology of early stage NSCLC had changed a lot in the past few years. Several recently published studies showed comparable OS between lobectomy and sublobar resection in stage IA NSCLC [12, 13]. The optimal surgical procedure for stage IA NSCLC remains controversial. Since the International Association for the Study of Lung Cancer (IASLC) lung cancer proposed to divide T1a into new T1a (≤ 1 cm) and T1b (>1 to 2 cm) in the eight edition TNM stage classification for lung cancer, a lot of attention has been focused on whether there is substantial difference in extent of lung resection for new T1a to T1b.

Beside extent of lung resection, whether LND is needed for early stage NSCLC is also controversial. Several randomized controlled trial compared survival between LND and lymph node sampling (LNS) [6, 7, 14]. Wu and colleagues suggested LND had obviously better survival compared with LNS for stage I NSCLC [6]. Sugi and colleagues showed no statistical significance was observed between LND and LNS in peripheral non-small-cell lung cancer less than 2 cm in diameter in their study [7]. ACOSOG Z0030 trial also showed mediastinal lymph node dissection does not improve survival in patients with early stage non-small cell lung cancer [14]. However, in ACOSOG Z0030 trial, mediastinoscopy was widely used and randomization was after negative mediastinal nodal sampling, so the results were not generalizable to patients staged radiographically. In study performed by Sugi et al. [7], the number of included patients is too small to achieve a valid conclusion.

In our study, we found segmentectomy had comparable OS and LCSS compared with lobectomy in NSCLC ≤1 cm and NSCLC>1 to 2 cm, which was contradict with previous study performed by Dai et al. [9] that also used SEER data. There are several reasons contribute to above results. Firstly, compared with previous study, more recently published SEER data were used in our study. In the past years, more and more ground glass-opacity nodules were detected, and several studies showed satisfactory survival were obtained after limited resection among these patients [15–18]. The different composition of histology may result in different survival status in our study. Secondly, extent of lymphadenectomy was not analyzed in previous study. Our study showed LND turned out to be an independent risk factor for better OS in patients with NSCLC ≤1 and better OS and LCSS for patients with NSCLC>1 to 2 cm. Without analysis of the status of lymphadenectomy, selection biases would inevitable exist in previous study and may explain the survival differences between two studies.

It is generally accepted LND can provide more accurate pathological stage for NSCLC when compared with non-LND. So, patients with non-LND may have more understaged patients than patients with LND, which may result in worse survival in non-LND group. Previous study concluded sublobar resection may result in more understaged lung cancers because of inadequate lymphadenectomy for hilar (N1) lymph nodes compared with lobectomy [19]. However, our study showed 61.1% patients in segmentectomy group, 75% patients in wedge resection group had less than or equal to 5 lymph nodes examined, which was even higher than lobectomy without LND group (55.4%) in NSCLC ≤2 cm. So the major reason for more understaged patients in sublobar resection group compared with lobectomy group in this study may attribute to lacking LND other than inadequate lymphadenectomy for hilar (N1) lymph nodes. After status of lymphadenectomy was analyzed in this study, no statistical survival difference was observed between segmentectomy and lobectomy. However, wedge resection still had worse OS in NSCLC ≤2 cm and worse LCSS in NSCLC>1 to 2 cm compared with lobectomy.

Compared with lobectomy, sublobar resection has the advantages of preserving better pulmonary function, fewer complications and lower mortality [20, 21], which is widely used in patients with NSCLC cannot tolerate a lobectomy due to compromised pulmonary function or advanced age. It is interesting to notice that the rate of segmentectomy (5.7%) is obviously lower than wedge resection (23.0%) and lobectomy (71.3%) in this study. More technically demanding than wedge resection and possibly worse survival than lobectomy may contribute to above situation. Since segmentectomy has advantages of better survival than wedge resection, preserving better pulmonary function and having comparable survival compared with lobectomy, segmentectomy should be encouraged to perform for patients with NSCLC ≤2 cm, regardless with or without compromised pulmonary function.

Compared with LNS, LND adds little morbidity to a pulmonary resection for lung cancer [22]. However, the impact on the operative process or postoperative course is limited. Our study showed obviously better OS and LCSS in lobectomy with LND group compared with lobectomy without LND group both in patients with NSCLC ≤1 and NSCLC>1 to 2 cm. Multivariable analysis suggested LND was independent risk factor for better OS in patients with NSCLC ≤1 and NSCLC>1 to 2 cm. Under this circumstance, LND should also be encouraged to perform for patients with NSCLC ≤2 cm, regardless extent of lung resection.

There were certain some limitations in this study. Given its retrospective nature, selection biases in treatment allocation were inevitable exist, although advanced statistical methods were applied in this study. Patients with limited cardiopulmonary function, elderly people were more likely to be allocated to sublobar group. However, the cardiopulmonary function situation was not given in our study, which was not able to balance by advanced statistical methods. Although comparing LCSS can exclude the influence of cardiopulmonary function situation in the maximum extent, this limitation could have a little impact on our results. Besides, we classified sublobar resection group as without LND group, which specific code was not provided in SEER database. Although the rate of examined lymph node less than or equal to 5 lymph nodes in sublobar resection was even higher than lobectomy without LND group, there was still a chance that small proportion of patients in sublobar resection received LND, which may have a little influence on our results.

## Conclusions

In conclusion, segmentectomy can achieve comparable survival compared with lobectomy in patients with stage I NSCLC ≤2 cm. LND can provide more accurate pathological stage, may affect survival, and should be recommended for above patients.

## Data Availability

Not applicable.
